# Child Fatalities in Tractor-Related Accidents in Rural Iceland, 1918–2024: A Historical Analysis

**DOI:** 10.3390/ijerph21101295

**Published:** 2024-09-28

**Authors:** Jónína Einarsdóttir, Geir Gunnlaugsson

**Affiliations:** Faculty of Sociology, Anthropology and Folkloristics, School of Social Sciences, University of Iceland, IS-102 Reykjavík, Iceland; geirgunnlaugsson@hi.is

**Keywords:** child, Scandinavian and Nordic countries, agriculture, farmer, accidents, off-road motor vehicles, mortality, premature, injury

## Abstract

Children on farms face high risks of work- and non-work-related fatalities, with tractors being a significant contributor. This study examines children’s involvement in fatal tractor-related accidents within agriculture in Iceland from 1918 to 2024, explores adult reflections on childhood tractor-driving experiences, and analyses Members of Parliament’s arguments against setting a minimum age for off-road tractor driving. The data are based on parliamentary debates on tractor-related legislation, fatal tractor-related accidents documented in newspaper archives and supplementary sources, and narrative interviews with former summer children who stayed at farms during their childhoods. Over half of the 81 registered accidents involved children—primarily boys—with 75% occurring between 1958 and 1988, when no minimum age for off-road tractor driving existed. The fatality incidence rate for children was more than four times higher than for adults. Arguments against minimum age requirements for off-road driving included the need for child labour, children’s superior driving skills, and the denial that children were more often victims than adults. Since 1988, no child has died while driving a tractor. A human-centred approach focusing on the working conditions, driver capacity, and adherence to safety procedures and legal frameworks is needed to prevent future accidents.

## 1. Introduction

Children raised on or staying at farms are subject to a risk of death related to work- and non-work fatalities, with tractors contributing to a large proportion of these deaths. Since the mid-20th century, vehicle-/tractor-related child fatalities in farming have been documented in various countries, including the United States [1,2,3,4,5,6,7], Canada [8,9,10], Australia [11,12], New Zealand [13], Sweden [14], Italy [15] Portugal [16], Spain [17], and Turkey [18,19]. However, the documentation of such incidents in other global regions remains limited [20]. Despite advancements in safety measures, tractor-related accidents in agriculture continue to be alarmingly prevalent, resulting in fatalities and injuries among both adults and children [21].

There are no reliable statistics on the direct impact of legislation regarding the minimum age requirement to drive a tractor on fatal accidents. In the United States, where driving a tractor is defined as a hazardous occupation, laws and regulations may differ among states [3,22]. Federal regulations do not allow children younger than 16 years to operate tractors or parts unless they work on farms owned or operated by their parents or have completed a certified tractor and machinery safety program. However, data indicate that removing this exemption would effectively prevent the most serious injuries among children working on their family farms [23]. In Sweden, children as young as 15 can obtain a licence to drive a tractor if they work on a family farm [24]. In the United Kingdom, children aged 13–15 can drive a low-powered tractor only after appropriate training [25]. As countries have increasingly enforced regulations regarding a minimum age for tractor driving and required training in their use, other measures have been taken, such as the introduction of rollover protective structures (ROPS), which have greatly improved the safety of operators [15,26,27].

Children and elderly male farmers are at the highest risk of tractor-related fatalities [6,10,14,18,19,28,29,30,31,32]. The tractor’s age is also important [17,28]. The primary types of fatal tractor accidents include rollovers, falls from tractors or attached equipment, runovers, crashes or collisions with motor vehicles, and entanglement in moving or rotating machine parts, particularly power take-off (PTO) shafts. While education, training, and safety equipment such as ROPS and seatbelts are widely recognised as crucial for accident prevention, legal measures are also recommended [4,26,27,32,33].

The adoption of tractors in agriculture was gradual in the United States, occurring from 1910 to 1940, influenced by the cost compared to horses, technological advancements, labour costs, and farm scaling [34,35]. The first tractor, an Avery model produced in the United States, arrived in Iceland in August 1918, and its price was equivalent to the value of the horses that it replaced [36]. The tractor only functioned properly on cultivated land, but a lack of knowledge hampered its use; e.g., it was initially only used in second gear. The importation of tractors was irregular in the following decades. The Association of Icelandic Cooperatives (SÍS) imported several dozen International 10/20 tractors between 1929 and 1931. Later, in 1944, the year when Iceland declared its independence from Denmark, 13 tractors of the Allis-Chalmers B model, so-called home tractors, were imported. One year later, 175 Farmall A tractors were brought in [37]. By 1960, partly due to the Marshall Plan approved by the Alþingi (the Icelandic Parliament) in 1948, tractors had become common property among Icelandic farmers [38,39].

The widespread adoption of tractors and various types of agricultural equipment transformed the labour demands in rural areas. Icelandic farmers highly regarded their tractors, often assigning them affectionate and humorous nicknames describing their characteristics [40]. However, the dangers associated with tractors in agricultural work quickly became apparent, particularly for rural children and the so-called “summer children”, i.e., urban children who stayed at farms during the summer months [41]. This practice of sending urban children to rural areas during the summer began in response to increased migration from rural areas to urban ones in the late 19th century. It was widely encouraged by state institutions, especially from 1950 to 1980, but continues to some extent today. The public held the custom in high regard and emphasised the importance of children learning to work while simultaneously enjoying nature and breathing clean, fresh air; it became part of being an Icelander [42].

In Iceland, until 1 July 1958, the legal driving age was 18 years for all vehicles on the main road and off-road. When the Traffic Act no. 26/1958 took effect, from 1 July 1958 to 29 February 1988, the legal age for the driving of cars and tractors on main roads was 17 years [41]. The minimum age to drive a tractor on the main road with a special license was 16 years, but there was no age limit for tractor driving off-road. Following debates in the Alþingi, the Traffic Act no. 50/1987 took effect on 1 March 1988. The legal age limit for tractor driving off-road became 13 years, and it increased to 15 years in 2006 to adapt to European legislation.

The primary objective of this study is to describe and analyse tractor-related fatal accidents in Icelandic agriculture, with a particular focus on children. Specifically, the aim is to address the following research questions: (1) To what extent have children been involved in fatal tractor-related accidents in Iceland since the introduction of tractors in 1918? (2) What are the reflections of adults who drove tractors in their childhoods during their summer stays on farms? (3) How did the tractor-related fatalities influence legislation on the minimum age for off-road tractor driving and the introduction of other safety measures?

## 2. Materials and Methods

The data presented here were collected as part of the research project “Independent Migration of Icelandic Children in the 20th Century Iceland” [42,43]. This project explored the Icelandic custom of sending urban children to stay at farms during the summer months.

### 2.1. Parliamentary Debates

The digital library *althingi.is*, which includes parliamentary debates, was searched using the keywords “dráttarvél” and “traktor” (both meaning “tractor” in Icelandic). Debates on legislation regarding a minimum age for off-road tractor driving and other preventive measures were analysed. The text was scrutinised for arguments for and against a minimum driving age and other accident prevention measures.

### 2.2. Fatal Tractor-Related Accidents

For this work, we repeated and expanded the search for fatal tractor-related accidents from the original research project to include data up to August 2024. We also reanalysed the data. A fatal tractor-related accident was defined as an incident resulting in death involving a tractor or equipment attached to a tractor (e.g., loaders, mowers, carriers, and PTO shafts). These accidents were identified through a comprehensive search on *timarit.is*, an open-access digital library of newspapers and periodicals from Iceland, the Faroe Islands, and Greenland.

The search strategy involved using the Icelandic words for tractor (“dráttarvél” and “traktor”) in combination with “slys” (accident). We compiled a database with all identified accidents and available information. Supplementary data were occasionally obtained through ad hoc contacts with relatives and subsequently confirmed using *timarit.is*. Commemorative articles, commonly published in newspapers, were analysed for additional details on victims and accident circumstances.

For Mac, the statistical analysis was performed using JMP Pro 16.0 (SAS Institute Inc., Cary, NC, USA). Chi-square tests assessed statistically significant differences (*p* < 0.05) in the proportions between groups. Incidence rates and odds ratios were calculated with 95% confidence intervals (CI). Incidence rates for fatalities in different age groups were based on population data from Statistics Iceland (*hagstofa.is*); a child was defined as a person younger than 18 years. The population in Iceland more than quadrupled from 91,368 residents in 1918 to 383,726 in 2024.

To calculate incidence rates, the definition of periods for tractor-related fatalities was guided by the enactment of two pieces of legislation on the minimum age to drive a tractor. In 1958, after enacting a law with no minimum age for tractor driving off-road, four tractor-related fatalities occurred; no tractor-related fatalities occurred in 1988 after the law on a minimum age of 13 years took effect.

### 2.3. Tractor Driving Narratives

Qualitative data were collected through narrative interviewing [44] between 2015 and 2017. While ensuring an even distribution across gender and age groups, the research group selected individuals who had stayed at a farm in their childhood [45]. Open-ended, semi-structured interviews were conducted with 52 individuals born between 1918 and 2001. The focus was on their experiences in driving tractors and their reflections on tractor accidents, which were widely discussed on the radio, in newspapers, and among the general population. All interviews were recorded, transcribed, and analysed using the Atlas.ti software (version 8.4.5, Berlin, Germany) [46].

### 2.4. Ethics

All participants in the qualitative component provided informed verbal consent before the interviews were conducted at locations of their choice (primarily their homes) and recorded with permission. Participants were informed about their right to anonymity and to withdraw at any time without giving a reason. The interviews and the anonymised transcripts were stored on the principal researcher’s password-protected computer. The research was approved by the Science Ethics Committee of the University of Iceland (15-005, 29 May 2015).

The use of secondary data on fatal tractor accidents and parliamentary debates, which are publicly accessible, did not require additional ethical approval. Given the sensitive nature of the data, care was taken to omit the names of individual Members of Parliament (MPs), accident victims, and specific accident locations.

## 3. Results

We present our findings in three subsections, i.e., parliamentary debates on legislation to prevent tractor-related accidents, the statistical analysis of tractor-related fatalities, and narratives from former summer children about their experiences in driving tractors during farm stays.

### 3.1. Parliamentary Debates on Tractor Use

The introduction of tractors in Iceland necessitated new legislation governing their use, including operational regulations. Frequent fatal tractor accidents were a primary concern, and parliamentarians debated prevention strategies. The main issues discussed included establishing a minimum age for tractor driving off-road for agricultural work and implementing other protective measures.

#### 3.1.1. Traffic Act No. 26/1958

On 18 February 1957, a new traffic law bill was submitted to the Alþingi. It proposed lowering the minimum age to drive vehicles on main roads from 18 to 17 years and for tractor driving off-road from 18 to 15 years [47]. Additionally, the bill proposed issuing a special licence allowing 16-year-old adolescents and adults without car driving licences to operate tractors on main roads. However, the bill’s sponsor argued upfront against the age limits for off-road tractor driving, suggesting no minimum age because such limits were likely to be disregarded [48].

The MPs debated the bill, disagreeing about the minimum age for off-road tractor driving [48]. Some noted that the Farmers Association favoured removing the age limit, proposing instead that tractor owners or operators should ensure that only capable individuals operated the machinery. During the 1957 and 1958 debates, MPs advocating for a law suggested a minimum age of 14 years for off-road tractor driving in farming and haymaking contexts. This group acknowledged children’s and adolescents’ desires to drive tractors but cited expert opinions that such activities could negatively impact neurological development without proper care and moderation. They argued that while a 14-year age limit would not entirely prevent accidents, older teenagers would likely be more capable of operating tractors than, e.g., 10 or 12 year olds. One MP emphasised that farmers should not rely on child labour for tractor operation and stressed the Aþingi’s role in implementing appropriate legislation to enhance agricultural safety.

In the spring of 1958, the Alþingi approved the Traffic Act no. 26/1958 [49]. This legislation stipulated that tractor drivers on main roads must possess either a car driving license or a special tractor license issued to individuals aged 16 years or older. However, it did not specify a minimum age for off-road tractor driving. MPs acknowledged that preventing tractor accidents would require an increased emphasis on education and safety measures, including ROPS.

#### 3.1.2. Debates on a Minimum Age Proposal in 1966

Following the Traffic Act no. 26/1958, a parliamentary proposal was approved on 27 March 1961 to investigate tractor accidents and recommend security improvements. Five years later, in the fall of 1966, a report revealed 23 tractor accidents since 1958 [50]. In response, an MP submitted a bill to amend the Traffic Act of 1958, proposing a 14-year minimum age for off-road tractor drivers and mandating the use of safety equipment [48].

The MPs debated the bill [48]. The Minister of Justice expressed concern regarding the fatal tractor accidents but questioned whether age restrictions would have prevented such incidents. He highlighted a recent regulation requiring importers to sell tractors with safety frames or cabs, which became effective in 1 January 1966. However, he noted that this regulation did not require farmers to retrofit older tractors with safety equipment. The Minister of Agriculture deemed the proposed 14-year age limit invalid, noting that older age groups experienced more accidents than adolescents.

Following parliamentary discussions, the Alþingi rejected the reintroduction of a minimum age for off-road tractor driving. The Farmers Association’s Annual Meeting had previously opposed such a requirement, arguing that fatal tractor accidents were equally distributed across age groups. Further, its members pointed out that age restrictions would limit farmers’ access to labour and instead proposed the stricter monitoring of tractor safety equipment.

#### 3.1.3. Debates on Safe Driving in 1970

In the fall of 1970, the MPs debated a proposal addressing tractor safety [48]. It suggested implementing regular inspections and considering whether farmers should be required to install safety equipment on older tractors [48]. The ensuing debate saw MPs generally supporting enhanced tractor security and driver education. However, some MPs cautioned against “expert” proposals that they deemed extreme, such as requiring lights on tractors only used during daylight hours. A few MPs expressed concerns about the high costs associated with tractor safety measures, while others argued that such expenses were negligible compared to the value of human life.

Although the proposal did not include minimum off-road tractor driving age provisions, some MPs argued against such restrictions [48]. One MP acknowledged the risks of allowing teenagers and children to drive tractors but maintained that age limits would not prevent accidents. Another MP, also a farmer, asserted that, in his experience, the most skilled tractor drivers were often 12–14 years old.

Following these deliberations, the Alþingi passed a bill instructing the government to promote the better supervision of tractor safety equipment and evaluate the need for enhanced driver skills.

#### 3.1.4. Traffic Act No. 50/1987

A traffic law bill submitted to the Alþingi in 1986 initially proposed setting the minimum age at 14 years for off-road tractor driving in agricultural work [48]. However, the MP sponsoring the bill on behalf of the General Committee of the Alþingi announced that the Committee recommended lowering this age limit to 13 years. The Committee viewed this reduction as “a more realistic approach for teenagers to use these devices legally” and believed that it would ensure that they received the necessary driving instruction. There was no debate, but one MP opposed the lowering of the age, noting that the “worst drivers” were typically 17–19. The bill was approved, and the Traffic Act no. 50/1987 came into force on 1 July 1988 [51], ending a three-decade period without age restrictions for off-road tractor driving in agricultural work.

### 3.2. Statistics on Fatal Tractor-Related Accidents

We identified 88 fatal tractor-related accidents from 1918, when the first tractor arrived in Iceland, to 31 August 2024. The first fatality occurred in 1948, when a tractor overturned with a young worker who was laying an electric cable on an uneven terrain. This accident and three others happened in workplaces outside the agricultural sector; three fatalities involved tractors on the main roads without links to farming activity. Excluding these seven fatalities, we identified 81 registered tractor-related fatalities within agriculture during 106 years of tractor usage in Iceland. Almost nine in ten deaths (88.9%) involved males, and more than half were children (Table 1).

#### 3.2.1. Incidence Rates

Most of the tractor-related fatalities occurred in the period 1958–1988 (Table 1); out of the 60 deaths in this period, 35 (58.3%) involved children. Across all age groups, the incidence in this period was 1.60 times higher than in 1950–1957 and 7.08 times higher than in 1989–2024 (Table 2). Similarly, the overall incidence of fatalities of children (<18 years), compared to that of adults, was 4.28 times higher in the period 1950–1957, 2.46 times in 1958–1988 and 1.58 times in 1989–2024. The highest incidence of fatalities occurred among children aged 12–17; it was 10.03 times higher than for adults in 1950–1957 and 4.42 times in 1958–1988 but was at the same level as that of adults in 1989–2024 (Table 2). After 1989, the incidence of tractor fatalities was more than seven times lower compared to 1958–1988, and none of the tractor-related fatalities involved a child driving a tractor.

#### 3.2.2. Positions of Victims

**Figure 1:**
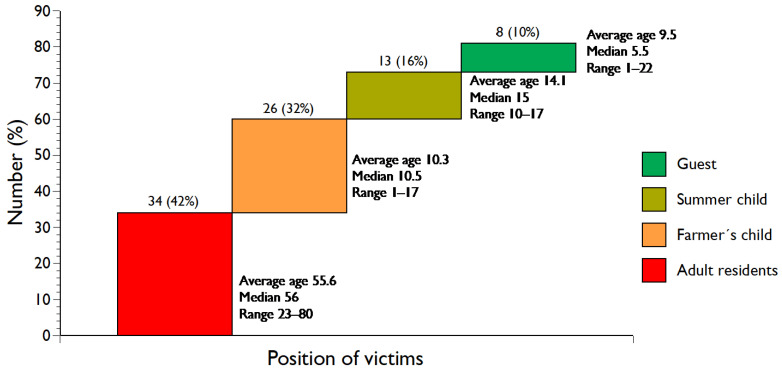
Positions of victims in tractor-related accidents and their ages (years).

Three quarters of the victims in tractor-related fatalities were farmers and their children (Table 1 and Figure 1). Nine (11.1%) fatalities involved females, i.e., one was a farmer, one was an adult child to farmers, five were young daughters of farmers, and two were guests. All of the summer children who died were boys.

Eight (9.9%) of the fatalities involved guests on the farm, namely six children and two adults (Table 1 and Figure 1); one of the adults (male) had previously been a summer child on the farm, who came to help with harvesting during a weekend. The guests were involved in rollover (two children, two adults), machinery (three children), and drive-over accidents (one child).

#### 3.2.3. Age at Death

The average age at death was 29.0 years (median 16, range 1–80); for males, it was 30.8 years (median 17, range 1–80), and, for females, it was 14.3 years (median 6, range 1–58). The difference in age between males and females was not statistically significant (*p* = 0.052).

Children (aged <18 years) were involved in 45 (55.6%) of the tractor-related accidents with a fatal outcome (Table 1); 38 (84.4%) were boys and seven (15.6%) girls. The mean age for boys was 11.6 years (median 13, range 1–17), compared to 6.1 years for girls (median 5, range 1–12); the age difference between boys and girls was statistically significant (*p* = 0.0060). The children of farmers were significantly younger than the summer children (10.3 years compared to 14.1 years (*p* = 0.0131)).

**Figure 2:**
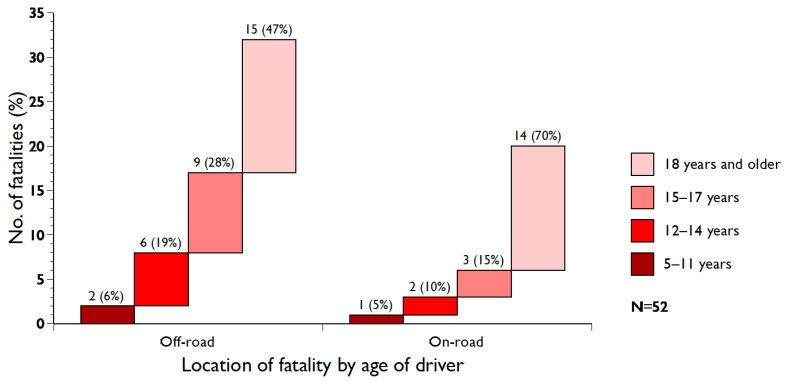
Age distribution of tractor drivers involved in fatal accidents and location of the accident.

Out of the 81 fatalities, 52 (64.2%) involved the driver of the tractor (Figure 2); their average age was 36.1 years (median 28, range 10–80). The average age of off-road drivers (*n* = 32) was 34.1 years (median 17, range 10–79), compared to 39.4 years (median 37, range 10–80) for those driving the tractor on-road (*n* = 20). There was no statistically significant difference (*p* = 0.4449) in the ages of those who drove off-road compared to those who drove on-road.

Out of 45 fatalities involving children, 23 (51.1%) were driving the tractor themselves (Figure 2). Their average age was 14.1 years (median 12, range 10–17). The majority of these fatalities (*n* = 20; 87%) occurred between 1958 and 1988; three occurred earlier, and no child has died while driving a tractor since 1988.

Twenty-nine (35.8%) fatalities involved passengers on the tractor or bystanders. Their average age was 16.2 years (median 10, range 1–78). All except one died in an off-road accident; 22 (75.9%) were children (<18 years). The mean age of the children was 10.8 years (median 12, range 1–17).

#### 3.2.4. Types of Accidents

**Figure 3:**
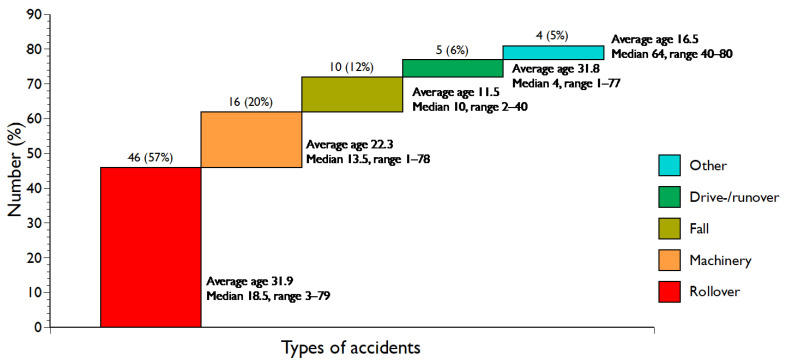
Types of tractor-related accidents and ages of victims (years).

Rollover was the most frequent type of accident, accounting for 46 (56.8%) out of 81 fatalities (Table 1 and Figure 3); all victims were male except one. Half of the rollover fatalities involved children, and 29 (63.0%) occurred off-road.

The average age of the 43 drivers in rollover accidents was 33.6 years (median 22, range 10–79); three non-drivers were aged 3, 7, and 15. In 20 (43.4%) out of 46 rollover fatalities, the driver was less than 18 years old; their average age was 14.6 (median 15, range 10–17).

Machinery-related fatalities were the second most frequent type of accident, accounting for 16 (19.7%) out of 81 cases (Table 1 and Figure 3). Fourteen (87.5%) involved males and 10 (62.5%) were children. All except one (93.8%) occurred off-road. Seven (43.8%) of the fatalities were caused by the PTO shaft of a tractor, and nine were caused by other types of machinery, e.g., hay carriages, mowers, and fertiliser spreaders.

Falls from tractors caused 10 (12.3%) fatalities (Table 1 and Figure 3), all occurring off-road. All except one (90%) were children, with five boys and four girls.

Other types of fatalities totalled nine and included five drive-/runovers (6.2%), two collisions (2.5%), one crushing (1.2%), and one (1.2%) caused by a snow avalanche; three (33.3%) were children. A drive-over involves a tractor with a driver, while, in a runover, the tractor runs over the victim without a driver.

**Figure 4:**
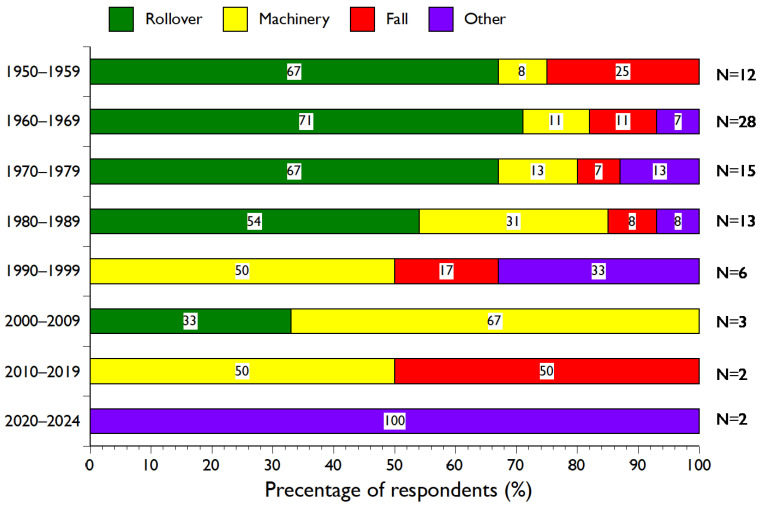
Types of tractor-related accidents with fatal outcomes since 1950, by number of fatalities (N) in 10-year intervals, except in 2020–2024.

The types of accidents have changed since 1950 (Figure 4). Rollover accidents were the most common from 1950 to 1989 but have largely disappeared, with the last one occurring in 2009. Machinery accidents continued across all decades, with the last one in 2011. Similarly, falls from tractors occurred occasionally throughout the whole period, with the last one occurring in 2015.

#### 3.2.5. Timing of Accidents

Most tractor-related fatalities occurred during the daytime and from April to September, i.e., during the work-intensive lambing and haying seasons (Table 1). There is no significant difference in the distribution across the days of the week, including weekends.

**Figure 5:**
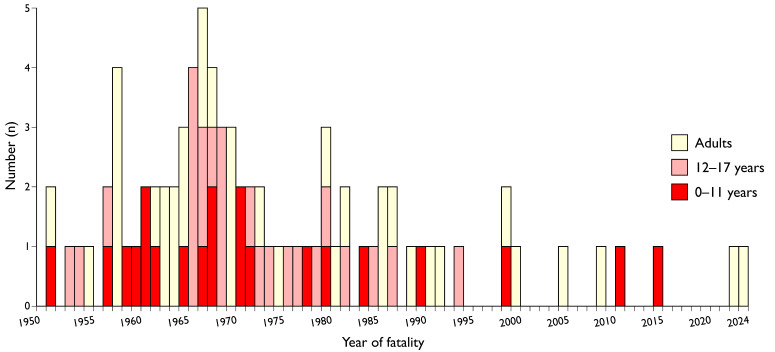
Tractor-related fatalities by year and age of victim from 1 January 1950 to 31 July 2024.

Figure 5 depicts the distribution of the 81 fatal accidents involving tractors from 1 January 1950 to 31 July 2024 by the victim’s age.

#### 3.2.6. Environment and Other Context

News reports often, but do not always, provide information about challenging circumstances at the time of the accident. Tractors traversed diverse terrain, including dirt roads, uncultivated fields, tussocks, marshlands, icy surfaces, and hills. Drivers lost control, sometimes with attached equipment, when crossing bridges of varying quality, navigating curves, and operating alongside ditches and rivers. Three news reports indicated that the driver causing the death of a bystander was a child or teenager; otherwise, no information was provided about the driver’s age. Weather conditions were sometimes adverse, and a snow avalanche hit one driver. Mechanical failure was cited as the probable cause in three accidents. Fatigue was reported as a factor for one young driver, while an adult operator was described as both intoxicated and exhausted.

### 3.3. Narratives about Driving Tractors: An Ambiguous Adventure

The narratives of the study participants who had stayed at farms during the summertime in childhood primarily focused on when and under what circumstances summer children began driving tractors, the tasks that they were entrusted with, and farmers’ awareness of safety issues. Above all, these accounts reflect the fascination that tractors held for many of these seasonal residents.

Tractors and farm machinery captivated summer children, particularly boys. A male participant who spent his summers in the countryside during the 1980s expressed his adoration for these machines and the joy of being “immersed in mud and machinery all day long” (Interview 14). He found the old, open tractors refreshing, noting no risk of falling asleep, unlike the newer models with enclosed cabs. Like most young drivers, he enjoyed the work. Some young drivers sang or listened to music while driving, simultaneously maintaining focus on the task at hand. Although fewer girls than boys drove tractors, some remembered the experience fondly.

A few participants did not express much enthusiasm for tractors. A male participant who spent his summers with his grandfather in the mid-20th century found driving tractors acceptable for travelling between points but did not engage in aimless driving (Interview 5). Another, who stayed at farms in the 1960s, initially found tractor driving exciting but grew weary due to the long working days (Interview 9). Some participants admitted that driving for extended periods was tiresome, and those of shorter stature recalled having to stand up from the seat to reach the clutch and brakes.

The age at which children started driving tractors varied considerably. One male participant began as young as 7 in 1982, although he considered his “real” start to have occurred at around age 11 or 12 (Interview 19). A female participant began operating machines at around 9 or 10, considering it part of the farm’s work ethic (Interview 46). Another did not recall her exact age but mentioned that her brother began at age 6 (Interview 44). Many children started at 9 to 12, often with simple tasks before progressing to more complex operations. The participants frequently noted that children raised on the farm began driving tractors earlier than they did and generally worked more.

The types of tractors and tasks assigned to children varied. Some drove older models without safety features. The newer tractors were described as heavy with enclosed cabs and roll bars; thus, children were often assigned to use the old ones, i.e., those without ROPS. Tasks included various haymaking steps, transporting goods, and even driving on public roads in some cases. The level of instruction provided to the children differed, with some receiving detailed adult guidance and others learning primarily through observation. A male participant who stayed at a farm in the 1980s mentioned that his uncle taught him extensively about tractors and cars, although safety was not explicitly discussed (Interview 16). A male teenager who drove a modern tractor in the 21st century received detailed instructions from a female farmer, whom he regarded as an expert driver (Interview 23). Sometimes, more experienced boys from the farm served as instructors. During the early years of tractor use in the countryside, farmers, some of whom had little experience in driving tractors, called upon experienced summer boys to assist with haying.

Safety was a concern, although the approaches varied widely. The narratives highlighted a lack of maintenance, which resulted in malfunctioning braking systems and steering mechanisms. Some farmers were, however, strict on maintenance and safety measures, while others seemed less concerned or lacked knowledge. All participants recalled hearing about tractor accidents on the radio, many of which were fatal. Several participants mentioned specific safety hazards, such as unprotected PTO shafts, which could catch clothing and cause severe injuries. As time progressed, the awareness of safety issues grew. A male participant staying at a farm in the late 1980s and early 1990s emphasised the strict safety measures implemented to prevent accidents (Interview 15).

The narratives indicated varying attitudes among parents and guardians regarding their children operating tractors and being around dangerous equipment. Some parents were fearful due to the known risks, while others seemed less concerned. An elderly male participant, who had stayed at a farm in his childhood before the tractor era, allowed his son to stay at a farm and drive a tractor despite his worries (Interview 1). His son’s classmate died in a tractor accident that same summer. There was a strong work ethic that sometimes prioritised productivity over safety, particularly during the haying season, when it was crucial to take advantage of sunny days. One male participant noted that tractor accidents were often viewed as “almost a natural sacrifice” within this work culture (Interview 9). Another participant recalled an incident when a tractor rolled over and the farmer died; in the afternoon, household members righted the tractor and continued working (Interview 28).

The farmers and summer children seemed well informed about the laws regarding tractor driving, particularly that children were not allowed to drive on main roads. However, they were unevenly concerned about compliance. All were aware of the frequently discussed tractor accidents; most recalled particular incidents. Nonetheless, only one male participant mentioned an incident involving police intervention (Interview 11). He had been stopped by police while driving a tractor on the main road, which he knew was unlawful. The situation was handled casually; his grandfather, unfamiliar with tractors and lacking a driving license, drove the tractor back to the farm. Afterwards, the police had coffee with his grandfather.

## 4. Discussion

This study examines fatal tractor accidents in Icelandic agriculture from 1918 to 2024, children’s narratives about tractors, and related legislation. Most fatalities involved adult farm residents, farmers’ children, and summer children who stayed at farms during the summertime. Most fatal tractor-related accidents occurred off-road; more than half involved children—primarily boys—and three quarters happened from 1958 to 1988, when legislation with no minimum age for off-road tractor driving existed. There was no age difference in fatalities between on-road and off-road drivers. Former summer children’s narratives provide insights into tractor use during farm stays and how tractors were received with admiration but also fear due to frequent reports of fatal tractor-related accidents. Meanwhile, MPs debated legalising a minimum driving age for off-road driving to prevent accidents. Arguments against a minimum age included the necessity of child labour, children’s purportedly superior driving skills, and claims that the child mortality rates were not higher than those for adults. Some suggested alternative measures like ROPS and improved education. Contrary to MPs’ claims, the results indicate that the incidence of fatalities among children was almost 2.5 times higher than that for adults from 1958 to 1988.

### 4.1. Children and Fatal Tractor-Related Accidents

Following Iceland’s independence in 1944, the import of tractors and the mechanisation of agriculture increased steadily. Farmers admired tractors for their capacity; however, most had limited experience in their usage. At the same time, the rapid rural-to-urban migration resulted in a lack of rural workforce. This shortage was partly alleviated by summer children, i.e., urban children who stayed at farms during the summertime and farm children who contributed to the rural economy with their labour [42]. Until the late 20th century, Icelandic children were also active in other industrial sectors, mainly fishing [53].

The findings highlight the high number of tractor-related fatalities in agriculture among children compared to adults; over half of all fatalities involved children (Table 1), and most victims were males. The incidence of deaths was more than four times higher among those aged 12–17 and 1.5 times higher for younger children aged less than 12 compared to adults when there was no minimum age for tractor driving off-road (Table 2). Furthermore, since 1988, when new legislation took effect establishing a minimum age of 13 years for off-road driving, the incidence of tractor-related fatalities in agriculture has substantially declined, with no fatalities among children driving tractors. As adult farmers are likelier to spend more time driving tractors than children, whose risk exposure is mainly during the summertime, the higher risk for tractor-related child fatalities than for adults is probably still higher than these results indicate.

Farming is recognised as a dangerous occupation [54,55,56], particularly for children [21,57], and the farm is characteristically the residence, site of play, and workplace for family members [6,56,58]. In Iceland, three quarters of all victims in tractor-related fatalities in the period 1918–2024 were the farmers themselves—almost entirely males and their children (Table 1 and Figure 1). Additionally, 16% of the fatalities involved summer children staying at the farm, a custom deeply rooted in Icelandic culture [42].

Rollover is recognised as the most common cause of tractor-related fatalities, followed by diverse machinery incidents [18,26,54]. Similarly, the rollover of tractors and associated machinery in Iceland caused three quarters of all tractor-related fatalities (Table 1 and Figure 3). One half of all rollover fatalities involved children, and, in more than two fifths of the cases, the driver was a child (<18 years). Research in Iceland on the practice of sending children to stay at a farm during the summer, based on a national random sample of adults, showed that almost half of all with the experience as children had driven a tractor during their stay on the farm; the mean age at the first time that they drove a tractor was 11.4 years, and one quarter drove a tractor before they reached ten years [41]. We do not have corresponding information about the farmers’ children, although we know from the narratives that they began driving tractors at a younger age than summer children. Their younger age further supports their younger age at driving a tractor at the time of death compared to the summer children (Figure 1), and almost half of them were driving the tractor.

In our data, children were caught in machinery, often at a young age, in 10 (62.5%) out of 16 such fatalities (Table 1 and Figure 3). In the US, from 1979 to 1985, more than half of all farm-related fatalities among children in two states involved tractors and other moving farming machinery [7]. Similarly, as seen in Turkey [18] and North America [4,7,29], the fatalities vary by season. In Iceland, most fatalities occurred from April to September, i.e., during the labour-intensive lambing and haying seasons (Table 1).

Both boys and girls are at risk of injury and fatality as bystanders and passengers within rural communities [54]. In our study, particularly young children, independent of gender, were at risk of death as bystanders and passengers. Three young children died in drive-over accidents: two girls and one boy. All ten falls from a tractor occurred off-road (Figure 2), and all victims but one were children. Guests are also exposed to the risk of injury while visiting the farm, irrespective of whether they come for play or work [58]. In our study, there were eight tractor-related fatalities involving guests (Table 1 and Figure 1). One of the adult guests was a former summer child.

Although children had a higher incidence of death than adults, the high number of fatalities among farmers is noteworthy. The former summer children’s narratives revealed that the farmers, particularly elderly ones, were not always experienced drivers, and the maintenance of the tractors could be deficient, with some of the tractors being old. The participants’ narratives highlighted how tractors were a recent phenomenon and often the first vehicle at the farm. The farmers had no experience in driving when they acquired their first tractor, and the roads were inadequate or non-existent, e.g., in the 1950s, a farmer died when his new tractor overturned on the way to the farm [41].

The allocation of tasks to children that they are not ready to perform due to their immature cognitive and judgment skills or physical strength is a recognised risk factor [21,54]. Driving a tractor was an exciting and ambiguous experience for the study participants. It could be challenging to drive a tractor, partly because the youngest drivers were too short and did not always reach the clutch and brakes. In addition, driving long distances was tiresome. All of the former summer children knew about tractor accidents when they stayed at the farm and a few experienced deaths or injuries among someone that they knew. As reported elsewhere, the farmers’ safety awareness varied, and this also applied to the children’s safety instructions [26,59,60].

### 4.2. Preventive Policy Measures

Most Icelandic MPs agreed to have no legalised minimum age for the off-road driving of tractors from 1958 to 1988. Their arguments aligned with three of five policy narratives used by policymakers in the Province of Alberta, Canada, to justify that child work within agriculture was unregulated [61]. The first one, that children’s agricultural chores are not real work, was not heard in the Icelandic Alþingi, nor did the Icelandic MPs emphasise the particularities of farming compared to other industries [48]. The MPs, who resisted legalising the minimum age for tractor driving off-road, underlined, like the policymakers in Alberta, that the farmers required children’s work. They also argued that education was more effective than regulation, that the families were better equipped to protect the children than any regulatory body. Furthermore, some MPs maintained that children were even better drivers than adults, and they rejected or ignored evidence that children were more likely to be victims of tractor-related accidents than adults. When confronted on the issue, the Icelandic MPs claimed that laws on a minimum age would not be followed anyway. These arguments have not stood the test of time, and, notably, in 1987, when the minimum age was discussed in the Alþingi, no MP repeated the former arguments or forwarded new ones against such a law [48].

The debates in the Alþingi illustrate the influence of the Farmers Association on legislation of importance for their interests [48]. At the time, most MPs had rural backgrounds and supported their constituencies. Whatever opinion MPs had on a minimum age to drive a tractor off-road, they agreed on the importance of education and other preventive measures. However, a few MPs warned against the “experts” exaggerating the safety demands and controls. In 1966, when ROPS became mandatory on new tractors, retroactive installation on older tractors was excluded because it would be costly for farmers, as found elsewhere [31]. However, the times are changing; no MPs opposed setting a minimum age of 13 years for off-road tractors at the Alþingi in 1988, when the number of fatal tractor-related accidents was already declining (Figure 5), and most of the fatalities after the legislation have been caused by machinery and falls (Figure 4). At that time, the farmers’ political position was weaker, and other legislation had been enacted that entailed policy to limit the overproduction of lamb meat and milk products [62].

Since 1988, in Iceland, no child aged less than 18 years has died when driving a tractor. Nevertheless, a direct association with the enactment of the Traffic Act no. 50/1987 is not justified. The number of fatal accidents was already declining along with the implementation of other preventive measures, including the increased use of ROPS; since 1988, one rollover fatality of an adult has occurred in 2009. Nonetheless, such accidents are still frequent elsewhere [26,27,33]. To further improve safety, drivers of ROPS-equipped tractors need to use seat belts, and passengers should not be allowed [15,54].

While formal education and training are crucial for the secure driving of tractors for all age groups, throughout the years, there have been calls for more regulatory approaches to prevent deaths and injuries among children within agriculture, including a minimum age for the operation of tractors and other machinery [2,57,63]. However, no research has confirmed the direct impact of such legislation. Researchers have instead argued for a minimum age to operate tractors and all-terrain vehicles due to the lack of capacity among children based on their physical, cognitive, social, perceptual, and sociocultural development [2,21,64]. The former summer children’s narratives presented in Section 3.3, who drove tractors in their childhoods, support these research findings. They also indicate that not all farmers were concerned about compliance with legislation and safety measures.

The circumstances and contexts of fatal accidents, as described in Section 3.2.6, might be seen as the ultimate cause of a deadly tractor-related accident. Fragnoli et al. [65] advocate for a human-centred approach to safety measures, emphasising the importance of addressing unsafe working conditions and the drivers’ capacity. They highlight the drivers’ need to anticipate potential hazards, maintain focus, and consider factors such as fatigue, stress, and the environmental conditions to enhance the overall safety. Based on the narratives of former summer children, a similar approach was warranted in Iceland; children and farmers alike worked long hours into the bright summer nights, and many highlighted how tiresome it was to drive a tractor all day long.

### 4.3. Strengths and Limitations

This study has several strengths. First, it is based on quantitative and qualitative data combined with secondary data on all tractor-related fatalities in Iceland from 1918 to 2024. Second, interviews with adults who stayed at farms during the summer in their childhoods highlight children’s fascination with tractors and how they drove tractors at a young age, with too many paying with their lives. Third, the debates of MPs in the Alþingi vividly illuminate the political power of farmers, who used child labour in increasingly mechanised agriculture and in times of rapid rural-to-urban migration.

This study has, however, limitations. First, information is lacking on who was driving the tractor in most cases when bystanders and passengers were the victims. Second, there is no information on other farm injuries, including accidents with fatal outcomes. Third, we might not have found all tractor-related fatalities with the search terms used, or the event may not have been reported in newspapers. Fourth, someone may have been injured in a tractor accident and died later without it appearing in the newspapers and annual public reports; however, this is unlikely considering the surveillance of tractor-related accidents by various public and private actors over the decades. Fifth, in calculating incidence rates, it is impossible to control for the time of exposure to a risk of fatality in tractor-related accidents in agriculture. There is also no information on whether the tractors were equipped with ROPS or other safety equipment at the time of the accident.

Finally, research on children’s experiences as tractor operators is lacking. Likewise, research is missing on contemporary arguments for the non-adoption of laws on a minimum age for tractor driving.

## 5. Conclusions

This study reveals that children have been at the highest risk of becoming victims of fatal tractor-related accidents in Iceland since the arrival of the first tractor to the country in 1918; they have lost their lives as drivers, bystanders, and passengers, most often off-road. It highlights the dangers of farming for rural children, other children staying at the farm, and child visitors.

While preventive measures, including education, ROPS, and the use of seat belts, are crucial, the resistance to setting a minimum age limit is remarkable; some of the MPs’ arguments against legalising a minimum age for tractor driving off-road do not stand the test of time. Our findings suggest that the abolition of the minimum age for off-road driving from 1958 to 1988 might have contributed to increased fatalities among children and adolescents, and probably also adults.

Legislators have a duty to alleviate obvious safety risks for their citizens, particularly for children. We highlight Lee’s [66] observation that referring to child farm injuries as “accidents” takes away adults’ responsibility; furthermore, it may impede the proper investigation of the incident and preventive efforts to secure children’s safety. Moreover, “in some cases, it denies justice to the child victim”. A human-centred approach to safety measures helps to address unsafe working conditions and environments, driver capacity, and adherence to safety procedures and legal frameworks to prevent future accidents.

## Figures and Tables

**Table 1:** Background characteristics of 81 tractor-related fatalities in agriculture in Iceland in the period 1918 to June 2024 involving children (<18 years) and adults.

Variable	Total		Children (<18 Years)		Adults	
	*n*	%	*n*	%	*n*	%
Age *						
0–4	7	8.6	7	8.6		
5–11	14	17.3	14	17.3		
12–14	9	11.1	9	11.1		
15–17	15	18.5	15	18.5		
18–39	9	11.1			9	11.1
40–59	15	18.5			15	18.5
60–80	12	14.8			12	14.8
Sex						
Male	72	88.9	38	46.9	34	42.0
Female	9	11.1	7	8.6	2	2.5
Position						
Farmer	32	39.5	0	0.0	32	39.5
Farmer’s adult son/daughter	2	2.5	0	0.0	2	2.5
Farmer’s child	26	32.1	26	32.1	0	0.0
Summer child	13	16.0	13	16.0	0	0.0
Visitor on farm	8	9.9	6	7.4	2	2.5
Cause of accident						
Rollover	46	56.8	23	28.4	23	28.4
Machinery	16	19.8	10	12.3	6	7.4
Fall	10	12.3	9	11.1	1	1.2
Drive-over	3	3.7	3	3.7	0	0.0
Runover	2	2.5	0	0.0	2	2.5
Collision	2	2.5	0	0.0	2	2.5
Crushing	1	1.2	0	0.0	1	1.2
Snow avalanche	1	1.2	0	0.0	1	1.2
Driver of tractor						
Yes	52	64.2	23	28.4	29	35.8
No	29	35.8	22	27.2	7	8.6
Road						
Off-road	60	74.1	39	48.1	21	25.9
Main road	21	25.9	6	7.4	15	18.5
Time period ^¥^						
1950–1957	7	8.6	5	6.2	2	2.5
1958–1988	60	74.1	35	43.2	25	30.9
1989–2024	14	17.3	5	6.2	9	11.1
Season						
Jan–Mar	10	12.3	5	6.2	5	6.2
Apr–Jun	14	17.3	7	8.6	7	8.6
Jul–Sep	45	55.6	29	35.8	16	19.8
Oct–Dec	12	14.8	4	4.9	8	9.9
Time of day						
Daytime (06–17)	61	75.3	35	43.2	26	32.1
Evening (18–23)	15	18.5	9	11.1	6	7.4
Night (00–05)	5	6.2	1	1.2	4	4.9
Weekday						
Monday–Friday	54	66.7	28	34.6	26	32.1
Saturday–Sunday	27	33.3	17	21.0	10	12.3

* We divide children into age groups in line with UNICEF for labouring children [52]. **^¥^** The Traffic Act no. 26/1958, with no minimum age for tractor driving off-road, was in effect from 1 July 1958 to 29 February 1988.

**Table 2:** Incidence rates of tractor-related fatalities in Iceland since 1950 in three periods.

	1950–1957			1958–1988			1989–2024		
Age (Years)	*n*	Incidence per 100,000 Person-Years	95% CI	*n*	Incidence per 100,000 Person-Years	95% CI	*n*	Incidence per 100,000 Person-Years	95% CI
0–4	1	0.64	−0.61–1.90	5	0.74	0.09–1.39	1	0.13	−0.12–0.37
5–11	1	0.57	−0.55–1.69	10	1.08	0.41–1.75	3	0.27	−0.04–0.58
12–14	2	3.37	−1.30–8.05	7	1.82	0.47–3.17	0	0.00	0.00
15–17	1	1.80	−1.73–5.33	13	3.52	1.61–5.43	1	0.21	−0.20–0.62
18–80	2	0.26	−0.10–0.62	25	0.60	0.37–0.84	9	0.11	0.04–0.18
All ages	7	0.57	0.15–1.01	60	0.92	0.69–1.15	14	0.13	0.06–0.19

## Data Availability

Data are contained within the article. The original contributions presented in the study are included, and further inquiries can be directed to the corresponding author.
